# Suicidal Risk in Women with Premenstrual Syndrome and Premenstrual Dysphoric Disorder: A Systematic Review and Meta-Analysis

**DOI:** 10.1089/jwh.2021.0185

**Published:** 2021-12-16

**Authors:** Divya Prasad, Bianca Wollenhaupt-Aguiar, Katrina N. Kidd, Taiane de Azevedo Cardoso, Benicio N. Frey

**Affiliations:** ^1^ Neuroscience Graduate Program, McMaster University, Hamilton, Ontario, Canada.; ^2^ Women's Health Concerns Clinic, St. Joseph's Healthcare, Hamilton, Canada.; ^3^ Mood Disorders Program, Department of Psychiatry and Behavioural Neurosciences, McMaster University, Hamilton, Canada.; ^4^ Biology & Psychology, Neuroscience, and Behaviour Honours Program, McMaster University, Hamilton, Canada.

**Keywords:** premenstrual syndrome, premenstrual dysphoric disorder, premenstrual symptoms, suicide attempt, suicidal ideation, suicidality

## Abstract

***Purpose:*** Women with premenstrual dysphoric disorder (PMDD) and premenstrual syndrome (PMS) experience substantial functional impairment and decreased quality of life. While previous research has highlighted a relationship between premenstrual disturbances and suicide risk, no meta-analysis has been conducted to quantitatively assess the findings.

***Methods:*** A systematic review and meta-analysis was conducted by searching the literature in three databases (Pubmed, PsycINFO, and EMBASE) on July 15, 2020. Studies that assessed the relationship between suicidality (attempt, ideation, and/or plan) and premenstrual disturbance (PMDD, PMS, and/or premenstrual symptoms) were included.

***Results:*** Thirteen studies were included in the qualitative review (*n* = 10 included in meta-analysis). Results revealed that women with PMDD are almost seven times at higher risk of suicide attempt (OR: 6.97; 95% CI: 2.98–16.29, *p* < 0.001) and almost four times as likely to exhibit suicidal ideation (OR: 3.95; 95% CI: 2.97–5.24, *p* < 0.001). Similarly, women with PMS are also at increased risk of suicidal ideation (OR: 10.06; 95% CI: 1.32 to −76.67, *p* = 0.03), but not for suicide attempt (OR: 1.85; 95% CI: 0.77 to −4.46, *p* = 0.17).

***Conclusions:*** Women with PMDD and PMS are at higher risk of suicidality compared with women without premenstrual disturbances. These findings support routine suicidal risk assessments for women who suffer from moderate-to-severe premenstrual disturbance. Furthermore, psychosocial treatments for women diagnosed with PMS/PMDD should consider and target suicidality to minimize risk and improve well-being.

## Introduction

Premenstrual symptoms affect up to 90% of menstruating women.^1^ While these symptoms are typically mild in nature, ∼30–40% of these women go on to experience premenstrual syndrome (PMS).^2^ This condition is characterized by moderate-to-severe physical, affective, and behavioral symptoms that occur within the luteal phase and remit once menstruation begins.^2^ Women with PMS may experience symptoms such as bloating, irritability, mood swings, social withdrawal, and poor concentration.^2,3^ Furthermore, ∼3–8% of women experience premenstrual symptoms of a greater clinical severity and are diagnosed with premenstrual dysphoric disorder (PMDD).^2,3^

Similar to PMS, symptoms of PMDD present during the luteal phase and disappear after menstruation commences.^2,4^ However, in contrast to PMS, PMDD typically confers marked functional and social impairment, severely affecting quality of life and well-being.^1,2^ Previously referred to as Late Luteal Phase Dysphoric Disorder in the third edition of the Diagnostic and Statistical Manual of Mental Disorders (LLPDD; DSM-III), PMDD is now categorized as a depressive disorder in the DSM-5^5^ and as a disease of the genitourinary system in the International Classification of Diseases update (ICD-11).^6^ Importantly, recent research has suggested that PMDD is the result of physiological shifts in reproductive hormones, and is associated with altered cellular processes and several neurobiological differences, such as abnormal dorsolateral prefrontal cortex functioning and executive control network connectivity.^7–9^

Given the considerable burden of PMS and PMDD on the functioning and overall well-being of women, both conditions are important topics for health research. This is especially true, given that the affective symptoms related to PMS and PMDD may render women vulnerable to suicidal tendencies, such as ideation, planning, or in severe cases, attempt. Indeed, previous literature has suggested that women with PMS and PMDD exhibit higher rates of suicidality when compared with women without these diagnoses.^10–12^ Interestingly, despite previous studies having uncovered relationships between PMS or PMDD and suicidality, there is still a paucity of research on these conditions compared with other psychiatric disorders.

Recently, Osborn et al. published a systematic review, finding that suicidal thoughts, ideation, plans, and attempts were significantly associated with PMDD.^13^ However, to our knowledge, no meta-analysis has quantitatively confirmed these results. To address this gap in the literature and expand upon previous findings, a systematic review and meta-analysis was conducted to explore whether premenstrual symptoms and/or diagnoses of PMS, LLPDD, or PMDD are associated with an increased risk of suicidality in women. These findings will contribute to the current knowledge base of PMS and PMDD, and may inform methods of screening, detection, and intervention that can improve mental health services available to women.

## Methods

The Preferred Reporting Items for Systematic Reviews and Meta-analysis (PRISMA) guidelines were utilized for the present review.^14^

### Protocol registration

This systematic review was registered in PROSPERO under the ID CRD42020199688.

### Search strategy

A literature search with no year or language restrictions was conducted on July 15, 2020, using the following databases: PubMed, PsycInfo, and Embase. Our search strategy was defined as: (“premenstrual syndrome” OR “PMS” OR “premenstrual dysphoric disorder” OR “PMDD” OR “late luteal phase dysphoric disorder” OR “LLPDD”) AND (“suicid*”).

### Inclusion and exclusion criteria

To determine whether an article was relevant to our study, we used the following inclusion criteria: cross-sectional, longitudinal, and case–control studies that (a) included women with premenstrual symptoms, PMS, LLPDD, or PMDD and compared their history of suicidal attempts, ideation, or plans with control women without any premenstrual disturbance; or (b) included women who attempted suicide and women who did not attempt suicide and compared their experiences of premenstrual symptoms, PMS, LLPDD, or PMDD. The exclusion criteria were as follows: (a) studies without comparison to controls without premenstrual symptoms, PMS, LLPDD, or PMDD; (b) reviews and meta-analyses; (c) case reports; (d) randomized controlled trials; and (e) abstracts, presentations, and editorials.

Studies were assessed by two blinded raters (D.P. and B.W.A.), who independently determined if studies met inclusion criteria by reviewing title and abstracts initially and full-texts later. Hand searches were also conducted to identify any articles that were previously missed in the database searches. Any discrepancies were resolved by seeking a third opinion (T.A.C.) to reach consensus.

### Data extraction

Two researchers (D.P. and K.K.) extracted data from all studies. A table was created to highlight the following aspects of each study: authorship, year, and country of publication, sample, study design, type of premenstrual disturbance assessed, assessment of suicidality (premenstrual symptoms, PMS, LLPDD, or PMDD), and main findings.

### Quality assessment

Each manuscript included was independently assessed by two blinded researchers (D.P. and K.K.) using the Newcastle-Ottawa Quality Assessment Scale (NOQAS) adapted for cross-sectional studies.^15^ Discrepancies were resolved by seeking opinions from the rest of the team (B.W.A. and T.A.C.), until consensus was reached.

### Statistical analysis

Random effects meta-analyses were performed using RevMan 5.4 software, and four analyses were conducted. PMDD diagnosis was analyzed as a risk factor for suicide attempt and suicidal ideation by comparing the number of cases in PMDD and non-PMDD groups to calculate the odds ratios. PMS diagnosis and premenstrual symptoms were analyzed as risk factors for suicide attempt and suicidal ideation, where the number of cases in the PMS/premenstrual symptom groups was compared with those in the non-PMS/no premenstrual symptom groups to calculate odds ratios. Significance was set to *p* < 0.05. Cochrane's Q test was performed to determine statistical heterogeneity, and the Higgins I2 statistic was utilized to assess the degree of variation between sample estimates (values range from 0% to 100%).

We contacted nine authors to determine or confirm the meta-analysis 2 × 2 table values. We received a response from six authors: four confirmed the data necessary for the 2 × 2 tables, one did not have the data available anymore, and one did not have data for groups separated by PMS, rather only by specific symptoms (and was thus excluded from the meta-analysis).

## Results

### Selection of studies

**FIG. 1.**
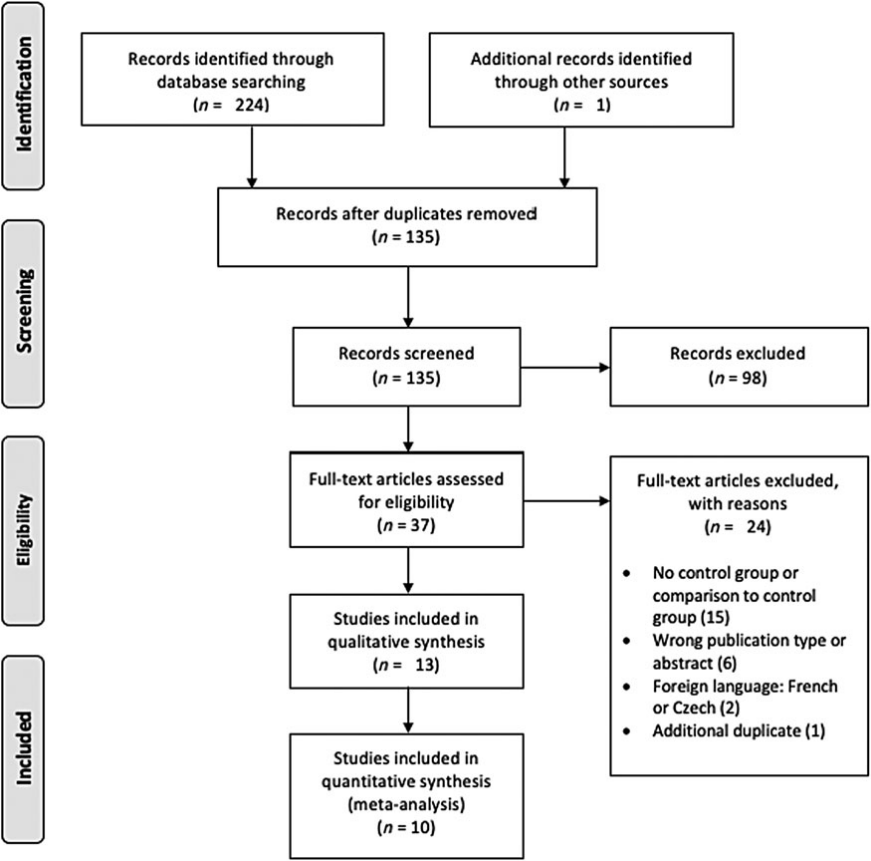
PRISMA flow diagram of studies included in the systematic review and meta-analysis.

The literature search yielded 224 studies, of which 90 were duplicates. Hand searches were also conducted to identify any studies that were previously missed (*n* = 1). A total of 135 articles were screened, leading to the exclusion of 98 articles based on publication type, study outcome, and/or design. The full-texts of the 37 potentially eligible studies were reviewed in full-text, and 24 were excluded based on the following reasons: no control group or comparison with a control group (*n* = 15), wrong publication type (*n* = 6), foreign language (*n* = 2), or additional duplicate (*n* = 1). Certain studies were excluded for more than one of the aforementioned reasons. In total, 13 studies were included in our systematic review and 10 studies in our meta-analysis (Fig. 1).

### Study characteristics

Among the 13 studies included, publication dates ranged from 1968 to 2018. Two studies were conducted in the United States, two in the United Kingdom, and one each in the following countries: Mexico, Spain, Germany, Turkey, Brazil, India, Republic of Korea, Malaysia, and Iran. Total sample sizes ranged from 68 to 3965 participants. All studies assessed the association between PMDD, PMS, or premenstrual symptoms and suicidality. No studies investigating LLPDD were identified in our search. Of the 13 studies included, seven evaluated associations between PMDD and suicidality, while nine evaluated the associations between PMS or premenstrual symptoms and suicidality. The characteristics of the studies included are described in Table 1.

**Table 1.** Data from Studies Included in the Systematic Review

Author, year, country	Sample	Study design	Type of premenstrual disturbance	Assessment of premenstrual disturbance	Assessment of suicidality	Main findings
Alvarado-Esquivel, (2018), Mexico^16^	*n* = 437 women aged 30–40 years	Cross-sectional	Premenstrual symptoms	Symptoms related to PMS were assessed through a face-to-face interview and a questionnaire.	History of suicide attempts and suicidal ideation was assessed through a face-to-face interview and a questionnaire.	Suicide attempts were found to be significantly associated with presence of irregular periods (OR: 9.57; 95% CI: 1.23–74.44, *p* = 0.03) and the desire to eat certain foods or eat a lot (OR: 3.08; 95% CI: 1.04–9.15, *p* = 0.04). Significant associations were also found between suicidal ideation and presence of irregular periods (OR: 2.89; 95% CI: 1.10–7.55, *p* = 0.03), low back pain (OR: 4.57; 95% CI: 1.31–15.92, *p* = 0.01), desire to eat certain food or eat a lot (OR: 2.47; 95% CI: 1.10–5.57, *p* = 0.02), guilty feelings (OR: 2.43; 95% CI: 1.06–5.59, *p* = 0.03), and aggressiveness (OR: 3.19; 95% CI: 1.42–7.13, *p* = 0.005).
Baca-Garcia et al. (2004), Spain^17^	*n* = 125 women who attempted suicide, *n* = 83 female blood donor controls	Case–control study	PMDD and PMS	Participants were asked about their menstrual cycles, and PMS and PMDD were assessed using DSM-IV criteria. The Mini-International Neuropsychiatric Interview (M.I.N.I. V 4.0) was used to make DSM-IV diagnoses.^18^	Women in the case group had recently attempted suicide, and this was used as the measure of suicidality.	There was a significant difference between the frequency of PMDD in the case and control group (54% vs. 6%, *p* ≤ 0.001). No significant differences were found in the incidence of PMS in suicide attempters with PMDD compared with controls with PMDD. Suicide attempters without PMDD had a comparable frequency of PMS as blood donors without PMDD.
Birtchnell and Floyd (1975), England^19^	*n* = 107 women who attempted suicide and *n* = 110 controls	Case–control study	Premenstrual symptoms	Participants completed a simple questionnaire inquiring about menstruation.	Women in the case group had recently attempted suicide, and this was used as the measure of suicidality.	No significant differences were found between the case and control groups in the incidence of premenstrual emotional disturbance (48.6% vs. 58.2% of case and control participants, respectively; *χ*^2^ = 1.460).
Chaturvedi et al. (1995), India^20^	*n* = 296 women aged 15–45 years	Cross-sectional study	Premenstrual symptoms	The Premenstrual Assessment Form (PAF) was used to measure distress during the premenstrual period.^21^	The PAF was used to identify participants who expressed suicidal ideas and death wish.	Women who reported suicidal ideas during the premenstrual phase significantly more often reported experiencing irritability, mood swings, sense of losing control, depression, and water retention during the premenstrual period (*p* < 0.001).
de Carvalho et al. (2018), Brazil^22^	*n* = 727 women aged 21 and 32 years	Cross-sectional study as part of a larger cohort study (“Psychosocial and Biological Factors in Bipolar Disorder: A Population-Based Cohort of Young Adults”).	PMDD	PMDD was assessed using the Mini International Neuropsychiatric Interview version Plus (M.I.N.I.-Plus), translated to Brazilian Portuguese.^18,23^	Current suicide risk was assessed through the M.I.N.I-Plus.	Women with PMDD were two to three times more likely to report suicide risk when compared with women without PMDD. Women with PMDD were at significant risk of current suicide (OR: 1.98; 95% CI: 1.33–2.96, *p* = 0.002).
Hong et al. (2012), Korea^24^	*n* = 2499 women aged 18–64 years	Cross-sectional cohort study as part of the larger Korean Epidemiologic Catchment Area (KECA) study	PMDD	The 12-month PMDD diagnostic module of the World Health Organization Composite International Diagnostic Interview (WHO-CIDI) was used to assess PMDD based on DSM-IV criteria.^25^	The Korean version of the Composite International Diagnostic Interview (K-CIDI) was used to assess lifetime and 1-year suicidality (ideation, plan, and attempt).^26^	Lifetime prevalences of suicide attempts (OR: 3.72; 95% CI: 1.71–8.10), suicidal ideation (OR: 3.99; 95% CI: 2.37–6.73), and suicide plans (OR: 4.43; 95% CI: 2.16–9.06) were significantly associated with PMDD once adjustments for age were made. Twelve-month prevalence of suicide attempts (OR: 6.17; 95% CI: 1.19–32.01), suicidal ideation (OR: 6.94; 95% CI: 3.45–13.96), and plans (OR: 7.29; 95% CI: 1.83–29.03) were significantly associated with PMDD as well. Once adjustments for psychiatric disorders were made, lifetime and 12-month prevalence of suicidal ideation were still significantly associated with PMDD (OR: 2.60; 95% CI: 1.47–4.61 and OR: 3.74; 95% CI: 1.70–8.24, respectively), while associations between PMDD and suicide attempt and plan did not hold.
Keye et al. (1986), USA^27^	*n* = 68 women presenting for evaluation of premenstrual complaints, *n* = 34 gynecology patients, *n* = 50 women seeking treatment at a Sex and Marital therapy clinic	Cross-sectional	Premenstrual symptoms	A detailed history of premenstrual complaints was taken from women accepted for the study. Participants also charted their symptoms on a calendar for 1 month, checking a box if a particular symptom was experienced. When possible, patients' husbands were also asked to record their daily behavior during the month.	The Minnesota Multiphasic Personality Inventory was used to measure emotional distress.^28^	75% of the women with premenstrual symptoms had thought of committing suicide compared with 17% of women from the gynecology clinic, while 21% of the women in the former group had attempted suicide in the past compared with 3% of the control group (*p* < 0.01).
Lee et al. (2006), Malaysia^29^	*n* = 2411 female students aged 12–19 years	Cross-sectional descriptive study	PMS	Participants were given a questionnaire that inquired about menstruation and PMS.	Suicidal behaviors (feeling sad or hopeless, seriously considering attempting suicide, and making a suicide plan and/or attempt in the past 12 months) were explored through the survey.	PMS was not significantly associated with suicide attempt (*p* = 0.440). A significant association was found between PMS and having seriously considered suicide (*p* = 0.007) as well as having made suicidal plans (*p* = 0.021).
Pilver et al. (2013), USA^11^	*n* = 3965 women aged 18–40 years.	Secondary data analysis of a subsection of participants from larger cohort study (Collaborative Psychiatric Epidemiology Survey)	PMDD and PMS	PMDD status (PMDD, moderate/severe PMS, or no premenstrual symptoms) was determined using the “Premenstrual Syndrome” module of the WMH-CIDI, based on DSM-IV criteria.^30^	Lifetime suicide attempt(s), ideation, and plans were assessed binarily. Participants read descriptions of behavior and relayed to the interviewer whether they had ever done the action. Positive response = 1, negative response = 0.	Women with PMDD were at increased risk of suicide attempt (OR: 2.10; 95% CI: 1.08–4.08), ideation (OR: 2.22; 95% CI: 1.40–3.53), and plans (OR: 2.27; 95% CI: 1.20–4.28) when compared with women with no premenstrual symptoms. Women with moderate-to-severe PMS had a greater likelihood of suicidal ideation (OR: 1.49; 95% CI: 1.17–1.88) in comparison with women with no premenstrual symptoms.
Shams-Alizadeh et al. (2018), Iran^10^	*n* = 120 women who had attempted suicide and *n* = 120 control women, all aged 13–40 years.	Case–control study	PMDD and PMS	Diagnosis of PMDD and PMS was obtained through a clinical interview led by an experienced psychiatrist, based on DSM-5 criteria.	Women in the case group had recently attempted suicide, and this was used as the measure of suicidality.	The case group had a significantly higher frequency of PMDD when compared with the control group (*p* = 0.001). No significant differences were found in the frequency of PMS between the case and control groups (*p* = 0.294).
Soydas et al. (2014), Turkey^31^	*n* = 70 outpatients with PMDD and *n* = 78 healthy controls, all aged between 18 and 40 years	Cross-sectional	PMDD	PMDD diagnosis was determined after a psychiatric examination and assessment through the Structured Clinical Interview for DSM-IV Axis I Disorders.^32^ The Premenstrual Syndrome Scale (PMSS) was used to measure the severity of PMDD symptoms.^33^ All participants had a psychiatric examination by a senior psychiatrist.	SCID-I, translated into Turkish.^32,34^	History of suicide attempt was found to be significantly higher in the PMDD group when compared with the healthy control group (*χ*^2^ = 27.78, *p* = 0.024).
Thin (1968), England^35^	*n* = 100 women who had attempted suicide (Group I), *n* = 57 women hospitalized with organic medical conditions (Group II), *n* = 68 healthy women (Group III), aged 15–44 years.	Cross-sectional	Premenstrual symptoms	Premenstrual symptoms were assessed using a questionnaire. Participants were requested to classify symptoms as “significant” (“present for at least one whole day before six or more periods during the preceding year”) or “insignificant.”	Women in Group I had recently attempted suicide, and this was used as the measure of suicidality.	The highest incidence of premenstrual symptoms was found in Group I (women who attempted suicide) when compared with Groups II and III (nonattempters; *p* < 0.01). Psychological premenstrual symptoms were found to be more common in Group I compared with Groups II and III.
Wittchen et al. (2002), Germany^12^	*n* = 1488 women aged 14–24 years	Prospective-longitudinal community study part of the Early Developmental Stages of Psychopathology study.	PMDD	Premenstrual syndromes were assessed using the PMDD module of the Munich-Composite International Diagnostic Interview, considering DSM-IV criteria.^36^	M-CIDI was used to assess suicide attempt and ideation.	Women with PMDD reported significantly higher rates of previous suicide attempts (OR: 4.4; 95% CI: 2.0–9.7, *p* < 0.001) when compared with non-PMDD control participants. However, no significant differences were found between suicidal ideation in the PMDD and non-PMDD group.

PRISMA, Preferred Reporting Items for Systematic Reviews and Meta-Analysis Guidelines; PMDD, premenstrual dysphoric disorder; PMS, premenstrual syndrome.

### PMDD diagnosis

Seven studies assessed PMDD through standardized clinical interviews based on DSM-IV or DSM-5 criteria.^5^ The following interviews were used: The Mini International Neuropsychiatric Interview (MINI 4.0 or MINI Plus, *n* = 2), Composite International Diagnostic Interview (Korean CIDI, World Mental Health CIDI (WMH-CIDI), Munich-CIDI, *n* = 3), or the Structured Clinical Interview for DSM-IV Axis I Disorders (SCID-I, *n* = 1). One study assessed PMDD through a clinical interview administered by an experienced psychiatrist for which additional details were not provided.^10^ PMDD diagnoses were considered provisional, as full diagnoses were contingent upon observation of two menstrual cycles.

### PMS diagnosis and premenstrual symptoms

Nine studies assessed PMS and/or premenstrual symptoms, with only a third using DSM-IV or DSM-5 criteria for diagnosis (*n* = 3). Pilver et al. utilized the WMH-CIDI to obtain PMS diagnoses based on DSM-IV criteria, and Shams-Alizadeh et al. conducted psychiatrist-led clinical interviews that were based on DSM-5 criteria.^5,10,11^ Similarly, Baca-Garcia et al. provided PMS diagnoses based on DSM-IV criteria.^17^ The remaining studies used the Premenstrual Assessment Form (PAF, *n* = 1), nonstandardized questionnaires and/or interviews (*n* = 4), or a calendar to chart symptoms (*n* = 1).

### Assessment of suicidality

Suicidality was split into three categories: suicide attempt, suicidal ideation, and suicidal plan. Of the 13 studies, 11 assessed suicidal attempts, 7 assessed suicidal ideation, 3 assessed suicidal planning, and 1 assessed suicidal risk—a composite measure of attempt, ideation, and planning. Results from this study are reported under the ideation section.^22^

Across the 13 studies, suicidality was assessed through a variety of measures, either using clinical interviews (*n* = 5), standardized questionnaires (*n* = 3), admittance to a hospital for suicidal attempt (*n* = 4), or binary symptom reporting in an interview (*n* = 1). Of the studies that utilized structured interviews, three used variations of the CIDI,^12,24,31^ while de Carvalho et al. used the MINI and Alvarado-Esquivel assessed suicidal attempt and ideation using a questionnaire-assisted interview.^16,18,22^ Of the studies that utilized standardized questionnaires, Chaturvedi et al. utilized the PAF; Keye et al. used the Minnesota Multiphasic Personality Inventory (MMPI); and Lee et al. used a self-administered structured questionnaire.^20,27,29^

#### PMDD and suicidality overview

The systematic review included seven studies that investigated the relationship between PMDD and suicidality. All studies found a significant association between PMDD and at least one category of suicidality, although two studies did not find support for an association between PMDD and every category of suicidality they assessed.

### Increased risk for suicide attempt in PMDD: evidence from observational studies

Six studies investigated the association between PMDD and history of suicide attempts or current suicide attempt.^10–12,17,24,31^ In total, five of six studies reported a significant association between PMDD and suicide attempts, while one study found a significant association in the crude analysis, but after adjustment for confounders (age and psychiatric disorders), the association did not remain significant.^24^ Among the six studies, four compared suicidality between women with and without a PMDD diagnosis, while the remaining two compared the incidence of PMDD in women who had attempted suicide (and were admitted to the emergency room) with controls.

Pilver et al. analyzed secondary data obtained from the Collaborative Psychiatric Epidemiology Survey.^11^ The sample included 3965 noninstitutionalized women aged 18–40 years. PMDD diagnoses were obtained using the “Premenstrual Syndrome” module of the WMH-CIDI.^30^ Suicidality was assessed by requesting participants to read a card describing a behavior and then inform the interviewer if they had previously engaged in this behavior. Responses were coded binarily, with positive responses corresponding to “1” and negative responses corresponding to “0.” Results indicated that women with PMDD were significantly more likely to report suicide attempts (OR: 2.10; 95% CI: 1.08–4.08, *p* < 0.05), when compared with women without any premenstrual symptoms.^11^

Wittchen et al. analyzed a sample of 1488 community women aged 14–24 years who had participated in the Early Developmental Stages of Psychopathology prospective longitudinal study.^12^ The PMDD module from the DSM-IV was used to diagnose PMDD. Results showed that women with PMDD reported significantly higher rates of previous suicide attempts (OR: 4.4; 95% CI: 2.0–9.7, *p* < 0.001) when compared with non-PMDD control participants.^12^ Soydas et al. conducted a cross-sectional study comparing 70 women who had been admitted to an outpatient psychiatry clinic and were diagnosed with PMDD with 78 healthy controls, where all participants were aged 18–40 years.^31^ The SCID-I was utilized to obtain PMDD diagnoses based on DSM-IV criteria and to assess suicidality. Results revealed that the PMDD group had higher rates of attempted suicide, when compared with the control group (*χ*^2^ = 27.78, *p* = 0.024).^31^

Finally, Hong et al. conducted a community-based cross-sectional study with a large sample of 2499 women between the ages of 18 and 64 years.^24^ The Korean-translated version of the 12-month PMDD module of the World Health Organization Composite International Diagnostic Interview (WHO-CIDI) was used to obtain PMDD diagnoses.^25^ Lifetime and 1 year suicidality were assessed using the suicide module of the Korean CIDI (K-CIDI).^26^ Results revealed significant associations between lifetime prevalence of suicide attempts and PMDD (OR: 3.72; 95% CI: 1.71–8.10, *p* < 0.01) after adjusting for age and between 12-month prevalence of suicide attempts and PMDD (OR: 6.17; 95% CI: 1.19–32.01, *p* < 0.05).^24^ However, once adjustments were made for psychiatric disorders as well, the associations between PMDD and suicide attempts did not remain.

Two studies compared women who had attempted suicide with nonattempter controls. Baca-Garcia et al. conducted a cross-sectional study with 125 women who had attempted suicide and 83 control women without a history of suicidal behavior or psychiatric disorders.^17^ While the authors did not provide an age range for participants, the mean age of the sample was 30.6 and 32.7 years for attempters with and without PMDD, respectively.^17^ The MINI 4.0 was used to assess PMDD. Results showed a significantly higher frequency of PMDD in women who had attempted suicide when compared with controls (54% vs. 6%, respectively, *p* ≤ 0.001).^17^

Shams-Alizadeh et al. conducted a case–control study involving 120 women who had attempted suicide and 120 women who were matched controls.^10^ Participants were aged 13–40 years, and PMDD diagnosis was obtained by a psychiatrist through a clinical interview based on DSM-5 criteria. Results revealed a significantly greater frequency of PMDD among women who had attempted suicide, compared with controls (*p* = 0.001).^10^

### Increased risk for suicide attempt in PMDD: results from the meta-analysis

We included four of the six studies mentioned above, in addition to one other study (de Carvalho et al.).^22^ One of the authors of this study is in our team and was able to provide data for lifetime suicide attempt of this study population. This information was taken into account in the meta-analysis. Raw data from the studies were used in all of the meta-analyses conducted in this review.

**FIG. 2.**
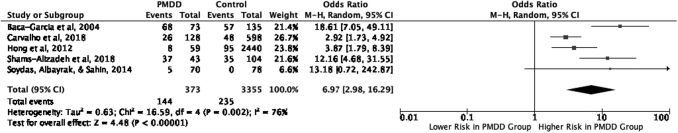
Meta-analysis of studies assessing PMDD and suicide attempt. PMDD, premenstrual dysphoric disorder.

The five studies included a total of 373 participants with PMDD and 3355 participants without PMDD. Figure 2 shows that PMDD diagnosis increased the risk of suicide attempt by approximately sevenfold (OR: 6.97; 95% CI: 2.98–16.29, *p* < 0.001). A sensitivity analysis was performed to compare studies that chose case and control groups based on PMDD diagnosis versus those that selected groups based on suicide attempt. Even when studies were separately analyzed, there was a significant relationship found between PMDD and suicidality.

### Increased risk for suicidal ideation in PMDD: evidence from observational studies

Four studies assessed the association between PMDD and suicidal ideation.^11,12,22,24^ Three of four studies showed a significant relationship between PMDD and suicidal ideation. Hong et al. found a significant association between PMDD and lifetime prevalence of suicidal ideation (OR: 3.99; 95% CI: 2.37–6.73, *p* < 0.001), even after adjustments for age were made.^24^ In addition, even greater significant associations were found between PMDD and 12-month prevalence of suicidal ideation (OR: 6.94; 95% CI: 3.45–13.96, *p* < 0.001).^24^ Once adjustments were made for psychiatric disorders, significant associations between PMDD and lifetime as well as 12-month prevalence of suicidal ideation were maintained.^24^

Similarly, results from Pilver et al. indicated that women with PMDD were significantly more likely to report suicidal ideation (OR: 2.22; 95% CI: 1.40–3.53, *p* < 0.001) than women without any premenstrual symptoms.^11^

In addition to the two studies mentioned above that support a significant relationship between PMDD and suicidal ideation, de Carvalho et al. reported a significant association between PMDD and a composite result titled “suicide risk.”^22^ This result considered attempt, ideation, and plan, and was computed during a cross-sectional analysis with a community sample of 727 young adult women aged 21–32 years. PMDD diagnosis and suicide risk were determined using the Brazilian Portuguese version of the MINI-Plus.^18,23^ Results showed that women with PMDD had higher rates of current suicide risk than control participants (OR: 1.98; 95% CI: 1.33–2.96, *p* = 0.002).^22^

While the previous three studies found significant associations between PMDD and suicidal ideation, Wittchen et al. did not find an association between PMDD and suicidal ideation.^12^

### Increased risk for suicidal ideation in PMDD: results from the meta-analysis

**FIG. 3.**
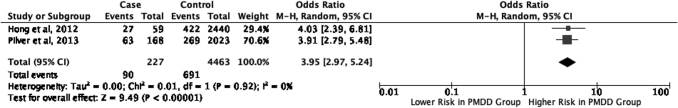
Meta-analysis of studies assessing PMDD and suicidal ideation.

We included two of the four studies mentioned above, including a total of 227 participants with PMDD and 4463 without PMDD.^11,24^ Our results (Fig. 3) showed that PMDD diagnosis increased the risk of suicidal ideation by approximately fourfold (OR: 3.95; 95% CI: 2.97–5.24, *p* < 0.001).

### Increased risk for suicidal plan in PMDD: evidence from observational studies

Two studies evaluated the association between PMDD and suicidal plan.^11,24^ One study reported significant associations between PMDD and suicidal plan,^11^ whereas the other found no significant association between these variables once accounting for confounding factors.^24^ Pilver et al. found that women with PMDD were significantly more likely to report suicidal plans (OR: 2.27; 95% CI: 1.20–4.28, *p* < 0.05) than women without any premenstrual symptoms.^11^ Hong et al. found a significant association between PMDD and lifetime prevalence of suicidal plans (OR: 4.43; 95% CI: 2.16–9.06, *p* < 0.001), after adjusting for age.^24^ Even greater significant associations were found between PMDD and 12-month prevalence of suicidal plans (OR: 7.29; 95% CI: 1.83–29.03, *p* < 0.001).^24^ However, once adjustments were made for psychiatric disorders, significant associations between PMDD and suicide plan were not maintained.^24^ Meta-analysis of results from this category was not possible, due to insufficient availability of data.

#### PMS and premenstrual symptoms overview

The systematic review included nine studies that investigated the relationship between PMS or premenstrual symptoms and suicidality. Five of nine studies reported a significant association between PMS/premenstrual symptoms and at least one category of suicidality.

### Increased risk for suicide attempt in PMS and with premenstrual symptoms: evidence from observational studies

Eight studies assessed the association between PMS and history of suicide attempt or current suicide attempt.^10,11,16,17,19,27,29,35^ Of these, three found a significant association between PMS/premenstrual symptoms and suicide attempt, while five reported no significant relationship between these variables.

Alvarado-Esquivel conducted a cross-sectional study of 437 menstruating women aged 30–40 years who had visited primary health care centers.^16^ PMS symptoms as well as the history of suicide attempts were assessed through a face-to-face interview and a questionnaire. Results revealed significant associations between history of suicide attempt and presence of irregular periods (OR: 9.57; 95% CI: 1.23–74.44, *p* = 0.03) as well as the desire to consume certain foods or eat a lot (OR: 3.08; 95% CI: 1.04–9.15, *p* = 0.04).^16^

Keye et al. investigated the association between premenstrual symptoms and history of suicide attempt in a sample of 68 women who responded to an article expressing medical interest in PMS and 34 controls from a gynecology clinic.^27^ The age range of participants was not provided. However, the mean age was 34.2 ± 0.42 years and 31.7 ± 0.96 years in the case and control groups, respectively.^27^ Differential diagnoses were recorded, and each participant was provided with a monthly chart to record symptoms. Results showed that 21% of women in the PMS sample had attempted suicide in the past, compared with 3% of controls (*p* < 0.01).^27^

Finally, Thin et al. assessed the premenstrual symptoms of 100 women who had attempted suicide and been admitted to a poison treatment center.^35^ These symptoms were compared with 57 women hospitalized for acute organic medical conditions and 68 women who were considered healthy. All subjects were aged 15–44 years. Results indicated that the greatest incidence of premenstrual symptoms occurred in the first group of women who had attempted suicide.^35^ When this incidence was compared with that of the two control groups, the difference was significant (*p* < 0.01).^35^

Five studies found no significant association between PMS/premenstrual symptoms and suicide attempt.^10,11,17,19,29^ First, Birtchnell and Floyd investigated the relationship between premenstrual emotional disturbance and suicide attempt in a sample of 107 female suicide attempters and 110 control women.^19^ Data regarding various aspects of menstruation were collected using a questionnaire that was given to all participants. Results indicated that there was no significant difference between the proportion of suicide attempters and control women who admitted to having premenstrual emotional disturbance (48.6% and 58.2%, respectively (*χ*^2^ = 1.460, df = 1)).^19^

Shams-Alizadeh et al. found no significant difference in the frequency of PMS reported by women who had previously attempted suicide versus controls (*p* = 0.294).^10^ Lee et al. studied the association between PMS and suicide attempt among 2411 female secondary school students, aged 12–19 years.^29^ Prevalence of PMS and history of suicide attempt were self-reported through self-administered questionnaires. Results indicated that PMS was not significantly associated with suicide attempts (*p* = 0.440).^29^

Baca-Garcia et al. reported no significant differences in the presence of PMS in suicide attempters with PMDD and controls with PMDD.^17^ In addition, there were no significant differences found in PMS in suicide attempters without PMDD and controls without PMDD.^17^ Finally, Pilver et al. found that after adjusting for psychiatric comorbidity, the association between moderate/severe PMS and suicide attempts was not statistically significant.^11^

#### Increased risk for suicide attempt in PMS and with premenstrual symptoms: results from the meta-analysis

**FIG. 4.**
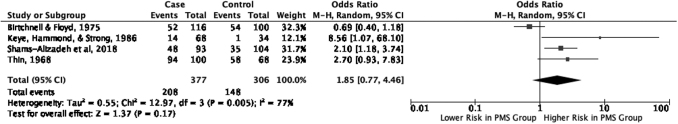
Meta-analysis of studies assessing PMS/premenstrual symptoms and suicide attempt. PMS, premenstrual syndrome.

We included four of the eight studies described above in our meta-analysis.^10,19,27,35^ These studies included 377 participants with a PMS diagnosis or premenstrual symptoms and 306 participants without a PMS diagnosis or premenstrual symptoms. Our results (Fig. 4) showed that there was no significant association between PMS and/or premenstrual symptoms and suicide attempt (OR: 1.85; 95% CI: 0.77–4.46, *p* = 0.17).

#### Increased risk for suicidal ideation in PMS and with premenstrual symptoms: evidence from observational studies

Five studies assessed the association between PMS or premenstrual symptoms and suicidal ideation.^11,16,20,27,29^ All of these studies found a significant association between PMS or premenstrual symptoms and suicidal ideation.

Chaturvedi et al. assessed the association between premenstrual symptoms and suicidal ideation in 296 women, aged 15–45 years.^20^ Premenstrual symptoms, as well as reports of suicidal ideation, were assessed through the PAF.^21^ Results showed that women who reported suicidal ideation during the premenstrual phase significantly more often reported experiencing irritability, mood swings, sense of losing control, depression, and water retention during the premenstrual period (*p* < 0.001).^20^

Alvarado-Esquivel also assessed the association between premenstrual symptoms and suicidal ideation, finding that suicidal ideation was associated with the presence of irregular periods (OR: 2.89; 95% CI: 1.10–7.55, *p* = 0.03), low back pain (OR: 4.57; 95% CI: 1.31–15.92, *p* = 0.01), desire to eat certain foods or eat a lot (OR: 2.47; 95% CI: 1.10–5.57, *p* = 0.02), feelings of guilt (OR: 2.43; 95% CI: 1.06–5.59, *p* = 0.03), and feelings of aggressiveness (OR: 3.19; 95% CI: 1.42–7.13, *p* = 0.005).^16^

Pilver et al. found that individuals with PMS were significantly more likely than controls to report suicidal ideation (OR: 1.49; 95% CI: 1.17–1.88).^11^ Keye et al. reported a significant association between PMS and suicidal ideation, with 75% of women with PMS reporting thoughts of committing suicide compared with 17% of the control population (*p* < 0.01).^27^ In addition, Lee et al. found that PMS was significantly associated with seriously considering attempting suicide (*p* = 0.007).^29^

### Increased risk for suicidal ideation in PMS/premenstrual symptoms: results from the meta-analysis

**FIG. 5.**
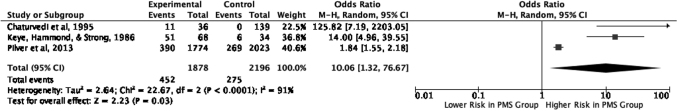
Meta-analysis of studies assessing PMS/premenstrual symptoms and suicidal ideation.

We included three of the five studies described above in our meta-analysis.^11,20,27^ These three studies included 1878 participants with a PMS diagnosis or premenstrual symptoms, and 2196 participants without a PMS diagnosis or premenstrual symptoms. Our results (Fig. 5) showed that women with PMS or premenstrual symptoms were at increased risk of suicidal ideation (OR: 10.06; 95% CI: 1.32–76.67, *p* = 0.03).

### Increased risk for suicidal plan in PMS and with premenstrual symptoms: evidence from observational studies

Two studies assessed suicidal plans in women with and without PMS.^11,29^ Lee et al. found a significant association between PMS and suicide plans (*p* = 0.021).^29^ In Pilver et al.'s study, significant associations between moderate/severe PMS and suicide plans disappeared once adjustments for psychiatric comorbidity were made.^11^ Meta-analysis of results was not conducted due to insufficient data availability in this category.

### Quality assessment

Table 2 shows the quality assessment for the studies included. Among the 13 studies, NOQAS scores ranged between 4 and 9. The mean NOQAS score obtained was 5.92/10, and the median score was 6/10. All studies except one^17^ failed to provide a sample size calculation and ∼50% had insufficient comparability between groups, due to inadequate control of other psychiatric disorders and remaining confounding factors. These two factors were the most common reasons for lower NOQAS scores across the studies reviewed.

**Table 2.** Newcastle-Ottawa Quality Assessment Scale Ratings

	Selection				Comparability	Outcome		
Authors	Representativeness	Selected group	Sample size	Diagnosis	Groups	Measurement method	Statistical test	Total
Alvarado-Esquivel (2018)	1	0	0	1	2	1	1	6
Baca-Garcia et al. (2004)	1	0	1	2	0	2	1	7
Birtchnell and Floyd (1975)	1	0	0	1	0	1	1	4
Chaturvedi et al. (1995)	1	0	0	1	0	1	1	4
de Carvalho et al. (2018)	1	1	0	2	2	2	1	9
Hong et al. (2012)	1	0	0	2	2	2	1	8
Keye et al. (1986)	1	0	0	1	0	1	1	4
Lee et al. (2006)	1	0	0	1	0	1	1	4
Pilver et al. (2013)	1	0	0	2	2	1	1	7
Shams-Alizadeh et al. (2018)	1	0	0	2	1	1	1	6
Soydas et al. (2014)	1	1	0	2	2	1	1	8
Wittchen and Pfister (1997)	1	0	0	2	1	1	1	6
Thin et al. (1968)	1	0	0	1	0	1	1	4

## Discussion

Results from our meta-analysis suggest that PMDD is a strong risk factor for suicidality, increasing the likelihood of suicide attempt and ideation by almost sevenfold and fourfold, respectively. In addition, our analyses indicate that women with PMS or premenstrual symptoms are also at increased risk of suicidal ideation, but not suicide attempt.

Our results are in line with a recent systematic review by Osborn et al., which found that PMDD is significantly associated with suicidal ideation and attempts.^13^ Our review also highlighted preliminary findings that PMDD may be associated with suicidal plans; however, current evidence is inadequate to make definite conclusions. In terms of PMS and premenstrual symptoms, our findings do not support a review by Saunders and Hawton, which concluded that there existed a significant association between PMS and suicide attempts.^37^ Rather, our review highlights that although women with PMS and premenstrual symptoms are at higher risk of suicidal ideation, only women with PMDD are at higher risk of both suicidal ideation and attempt.

While previous reviews have explored suicidality in relation to PMDD, PMS, and/or premenstrual symptoms separately, the present review provides a unique perspective by considering associations between all three groups and suicidality. Moreover, this review includes both a qualitative assessment and quantitative meta-analysis, the latter of which has not been previously conducted in this area, to our knowledge.

Our findings may be best explained by highlighting the special affective qualities and traits that are characteristic of PMS and PMDD. Previous research has shown that women with PMS and PMDD experience significant emotional dysregulation. For instance, Petersen et al. found that women with PMDD reported significantly greater scores on the Difficulty in Emotion Regulation Scale (DERS) when compared with controls.^4^ Similarly, Wu et al. found a negative association between the severity of PMS and habitual use of reappraisal, an emotional regulation strategy tied to the experience of greater positive emotion, improved well-being, and more optimal functioning.^38,39^ On the contrary, they noted that severity of PMS was positively associated with the habitual use of suppression, an emotional regulation strategy implicated in the experience of greater negative emotion, poorer well-being, and less optimal interpersonal functioning.^38,39^ These findings indicate that both PMS and PMDD are associated with emotional dysregulation, and that women with PMDD are likely more susceptible to using poorer emotion regulation strategies to cope with life stressors.^38,39^

While struggling to manage their emotions, women with PMS and PMDD also experience difficulties in impulse control. Ducasse et al. found that within a sample of women who had attempted suicide, those with PMS or PMDD were more impulsive and aggressive.^40^ Likewise, Petersen et al. found that women with PMDD reported higher scores on the behavioral impulsivity subscale of the Barratt Impulsivity Scale (BIS-11), when compared with controls.^4^ Previous research has linked impulsiveness and aggressiveness to an increased susceptibility to suicidal behavior, providing some explanation as to why, especially when compounded with emotion dysregulation, women with PMS and PMDD may be at greater risk of suicidality.^41^

Furthermore, women with severe PMS/PMDD may be vulnerable given their impaired interpersonal functioning, which may hinder relationships and lead to feelings of isolation and loneliness. Previous research has shown that women with PMDD report lower levels of social connectedness in comparison with healthy controls, and that in a sample of women with PMS, the severity of premenstrual affective symptoms is significantly associated with social impairment.^4,42^ Consequently, the interplay between affective disturbances, poor impulse control, and decreased support from interpersonal relationships may contribute to a suicidal risk profile for women with PMS and PMDD.

## Limitations of This Review and Included Studies

Our results should be interpreted in light of the following limitations. Authors screened studies written in English and Spanish only, thus excluding other studies that may have allowed for a more comprehensive analysis. Furthermore, all included studies except one featured a cross-sectional design, restricting any ability to draw causal relationships beyond a positive association between PMDD, PMS, premenstrual symptoms, and suicidality. Within the studies, various assessment methods were utilized, including nonstandardized and self-report measures, making it difficult to cross-compare and objectively review the results. Provisional diagnoses of PMS and PMDD within studies may have also led to a misrepresentation of these conditions in study participants and an overestimation of the reported prevalence.

The results of the meta-analyses were also limited by two major factors. First, since the included studies involved a wide range of sample sizes (68–3965), results were dominated by the larger studies involving more participants. Second, certain studies were excluded from the meta-analyses if data were unclear or missing from the original publications and author confirmation was not possible. This likely led to final estimates that were not wholly representative of all studies in a particular category, such as in the meta-analysis of PMDD and suicidal ideation, where the only study that did not support a significant association could not be included due to a lack of author confirmation.^12^

As for quality assessment, the ratings were modest (mean = 5.92/10) due to common limitations. For instance, all studies in the present review opted for convenience sampling instead of random sampling, and many studies did not control for psychiatric comorbidities. Despite these limitations, our review presents a comprehensive assessment of current cross-cultural PMDD and PMS research, offering a holistic view of how premenstrual disturbances are linked to suicidality. Our review also includes a meta-analysis, thereby providing quantitative results, which has not been previously done.

## Implications for Clinical Practice and Future Directions

Currently, recommendations for women with PMS/PMDD include pharmacotherapy, psychotherapy, lifestyle modification, and vitamin and herbal supplements.^43^ The first-line intervention for PMDD is pharmacotherapy, specifically the administration of serotonin or serotonin- and norepinephrine-based antidepressants.^43^ In terms of psychotherapy, cognitive behavioral therapy (CBT) has been investigated through a systematic review by Lustyk et al., and the findings showed that CBT was less effective than pharmacotherapy in treating PMDD.^44^ While psychotherapy may be useful as adjunctive to pharmacotherapy, the importance of medical treatments for PMDD cannot be understated. Future research should explore multidimensional treatment approaches for individuals with severe premenstrual disturbances, whereby pharmacotherapy and psychotherapy can complement one another to potentially reduce suicidal risk.

## Conclusion

Our systematic review and meta-analysis show that women with PMDD are at increased risk of suicidal attempt and ideation. This review also suggests that women with PMS experience a higher risk of suicidal ideation, but not suicidal attempt. Health care professionals should be aware that premenstrual disturbances of any kind, particularly those of increased severity such as that observed in PMDD, may confer suicidal risk for women. Thus, routine clinical examinations should assess suicidality to identify women with suicidal thoughts or tendencies, and guide them toward appropriate treatment resources. Research examining the effectiveness of psychosocial treatments targeting emotional regulation and suicidality in women with severe PMS/PMDD is highly encouraged.
